# A Criticism of Homœopathy

**Published:** 1909-04-24

**Authors:** C. E. Wheeler

**Affiliations:** 5 Devonshire Street, Portland Place, W.


### Editor's Letter-Box.

### "A CRITICISM OF HOMŒOPATHY."

*To the Editor of* The Hospital.

Sir,—Homœopathists, remembering past days, should perhaps be grateful to you that your article under this heading is written "more in sorrow than in anger"; but had you studied the subject more profoundly you would have avoided one or two errors. You deny that vaccine therapy is "of the essence" of homœopathy. Well, vaccine therapy consists in treating diseases with substances derived from the very bacilli that cause these diseases, and if that is not treating "likes with likes" then language has no meaning. You are in a position similar to those who maintain that municipal ownership of gas or trams or water is not as the essence of Socialism. It is not the *whole* of Socialism; it may be held desirable by men who do not accept the whole doctrine of Socialism; but it *is* Socialism as far as it goes, and rightly so called by those who care to call things by their names.

In the endeavour to escape from the position you (no doubt in ignorance) misrepresent the attitude of the homœopathist towards "symptoms." Let me endeavour to make it clear. The aim of the homœopathist is the aim cherished and recommended by all good clinical teachers—to treat the individual patient. He holds that the symptoms and physical signs of any given case are the result of an agent or agents acting on an individual constitution, of a certain seed, as it were, growing on a certain soil, and that to neglect either seed or soil is to fail in the best way of dealing with a condition which is the result of both. From the *whole*—the totality—of the symptoms and signs he shapes to himself the picture of his case. Now, drugs in their turn produce symptom-complexes comparable to the symptom-complexes of disease. The contention of the homœopathist is that the drug which has produced a symptom-complex most resembling the picture of his given case is the desirable remedy for that case, and the search for the application of that remedy is the practice of homœopathy. Therefore the homœopathist applauds the vaccine therapist who distinguishes one kind of cystitis from another. That is individualisation, and is the only road to successful treatment. He knows, indeed, that the vaccine therapist has already found that he must go further and recognise that (say) a staphylococcus vaccine derived from cocci of case A will not necessarily cure a staphylococcal disease in case B, so individual are our cases. Similarly, the homœopathist looks for indications for a remedy for his particular case, not for a remedy for the disease in general. Now the accuracy of this contention of the homœopathist is held by him to depend on the results of his daily experiences with remedies. It is, in fact, a matter for experiment and experience. Any man who will be at pains to master a little drug symptomatology can test the principle of homœopathy for himself, and homœopathists justly refuse to regard as scientific the procedure of denying their conclusions without testing their evidence. For instance, we all know that cantharides can produce an acute nephritis. Now recently a French doctor (not a homœopathist) has announced that drop doses of tincture of cantharides are very beneficial to cases of acute nephritis. (N.B.—The discovery has been made more than once before.) Now any man can try this for himself. If his experience is successful he will have proved, in one instance at least, that the like can benefit the like, and may be induced to extend his researches; if, on the other hand, he fails to establish a curative relation between drug and disease, his results, carefully recorded, will be a practical contribution towards a problem which can be settled by prolonged and painstaking experiment better than by *à priori* argument.

Yours faithfully, C. E. Wheeler.

5 Devonshire Street, Portland Place, W., April 16.

